# Effects of Antibiotics on Dental Implants: A Review

**DOI:** 10.4021/jocmr658w

**Published:** 2012-01-17

**Authors:** Nabeel Ahmad, Najeeb Saad

**Affiliations:** a Schulich School of Medicine & Dentistry, University of Western Ontario, Canada; b Assistant Director-Clinical Affairs, Chair-Restorative Dentistry, Schulich School of Medicine & Dentistry, University of Western Ontario, Canada; c Imaging Research Laboratories, Robarts Research Institute, London, Ontario, Canada; d Medical Biophysics, The University of Western Ontario, Canada

## Abstract

There are many reasons for dental implant failure, the development of bacteremia is concern for dentists. This is due to the possibility of unfavorable result such as implant loss or the need for re-treatment. In general, antibiotic prophylaxis is recommended for high risk patients such as individuals with an immunodeficiency, infectious endocarditis, or previous prosthetic instrumentation. However regarding clinically healthy, low and moderate risk individuals the use of antibiotics in implant dentistry is controversial. Another major concern regarding the over prescription of antibiotics is the selection of antibiotic resistant bacteria. A better understanding of the efficacy of prophylactic treatments regarding dental implantation is crucial. Thus a literature review and comparison of survival rates of dental implants with regimens of no, pre or post prophylaxis was performed using systematic literature review using reliable electronic databases. Retrospective or prospective controlled studies were examined for the influence of preoperative and/or postoperative or no antibiosis on dental implant success rate. Of the 11406 implants used in this literature review, cases with no antibiotics had a 92 % success rate, cases with pre-op antibiotic alone had a 96% success rate, cases with post-op antibiotic alone had a 97% success rate and cases with both pre and post-op antibiotic had a success rate of 96%. Thus, the results from this literature review show a > 90% success rate when antibiotics are used compared to when they are not used. Thus, no benefit is seen from the use of antibiotic prophylaxis in low and moderate risk dental implant patients.

**Keywords:**

Dental implants; Pre-operative prophylactics; Post-operative prophylactics; Success rate

## Introduction

Dentists and other physicians are often faced with the decision of whether to prescribe prophylactic antibiotics for complex oral surgeries such as dental implants. Although practitioners generally make these decisions on a case by case basis, if antibiotics were to be improperly prescribed it would produce a greater difficulty in treating legitimate infection [1]. Interestingly, a study done in 2000 revealed that 40% of dentist would prescribe antibiotics to patients with no relevant medical history as a contingency for infection [2]. This is of concern because according to the Canadian Dental Association (CDA) dental procedures, including implants, have become a common procedure and are on the rise [3]. Therefore, it is crucial that an appropriate case specific guideline is available for practitioners on the use of prophylactic antibiotics.

Antibiotics are used to prevent infection if a dental implant does become infected the chances of failure are high [4]. Though a number of factors can ultimately lead to the failure of dental implants, most practitioners take extra precautions regarding infection [5]. With the mouth being an inherently ”dirty field“, with a multitude of flora, the incidence of bacteremia is also high. The aim is to prevent the onset of infection in the surgical wound by achieving an antibiotic concentration in the blood that will prevent bacterial proliferation and dissemination [6].

Early implant failure is commonly associated with certain strains of bacteria. The most common bacteria involved in these types of infections include: streptococci, anaerobic Gram positive cocci, and anaerobic gram negative rods [7-11]. Thus, the antibiotic of choice for the prevention of delayed wound healing should be bactericidal and of low toxicity [12].

The American Heart Association recommends Amoxicillin and Penicillin as a first line of treatment due to their superior absorption and prolonged serum levels. However, in today’s population there is an increased level of penicillin allergies, thus a good alternative is clindamycin [13]. The use of antibiotics in implant dentistry is controversial. With the administration of antibiotics adverse events may occur, ranging from diarrhea to life threatening allergic reactions [14]. Major concerns associated with the widespread use of antibiotics is the evolution of antibiotic resistant bacteria [15], along with the routine use of antibiotics may lead to lax surgical techniques and actually increase the rate of complications [16].

Recent trends have explored the routine administration of antibiotic prophylaxis and look into the clinical research to substantiate its use. For decades, the use of antibiotics during dental surgical procedures has been condoned by major associations. According to the CDA, ”all dental procedures where significant oral bleeding and/or exposure to potentially contaminated tissue occurs typically (will) require antibiotic prophylaxis“ [3]. The American Dental Association (ADA) also suggests similar guidelines [17]. In addition, the American College of Surgeons and the American Heart Association (AHA) guidelines [18] suggests that complex oral surgery, including implant placement, will benefit from prophylactic antibiotic coverage; however, as of 2007, these associations have noted more leniency regarding dental implants and prophylaxis. They currently suggest that only high and some moderate risk category patients should receive antibiotics. Other concerns mentioned are that patients with previous prosthetics should be given extra caution, due to the increased probability of developing a bacterial infection [19].

The pre- or postoperative uses of antibiotics in combination with dental implant surgery and success rates are poorly documented in the current literature. Pre-operational standard guidelines regarding antibiotics are possible to create; however, post-operational may remain open-ended and can be based on procedural outcomes during and after completion of the operation. Unfortunately, although multiple articles have been written, double blind control trials remain lacking because of ethics.

The purpose of this paper is to review the current literature and information on dental implants and prophylaxis. Our objectives are to ask whether or not antibiotics are beneficial to implants, and in what instances pre- and/or postoperative antibiotic regimes should be prescribed. Hence we attempted to review the multitude of case series in literature to decipher this issue in clinical practice.

## Methods

The systematic literature review was completed using the electronic databases, Pubmed, Medpilot and Medline. Main search terms were antibiotic, prophyla combined with dental implant, implant failure, osseointegr and oral implant. Studies which met the inclusion criterion were English studies conducted between 1955 to January 2009 and which were retrospective or prospective controlled studies examining the influence of preoperative and/or postoperative or no antibiosis on dental implant failure rates. Administrations of various prophylactic antibiotics regimens were accepted. An unsuccessful dental implant was characterized by any implant which failed within the first 3 months, and studies with follow ups within the first 5 months were included. Studies with loading were not included and only studies using low risk patients were included. Of the 853 titles and abstracts which were identified by the literature search simply by word association, 797 were excluded due to either having no relevant material to implants and success rates regarding antibiotics, or were not prospective or retrospective studies. Fifty-six were selected for detailed review due to a relevant title. The next 45 titles were not acceptable because while relevant did not provide information on the antibiotic regimens prescribed, failed to provide a proper timeline of the implant procedure and/or were unclear or not specific enough to draw conclusive patient data. Among these, six met the criteria stated above and were included in our review (Table 1).

**Table 1:** Patients Data from Liturature

**Primary author**	**# of patients**	**# of implants**	**Intervention**	**Other allowed interventions**	**Implant survival (following 3 months)**
Binahmed^19^	125	445	1 hour post-op: 1,000,000 units penicillin	0.12% chlorhexidine pre-	445/445
			intravenous or equivalent oral doses of	and postoperative mouth rinses	
			penicillin V or 600 mg clindamycin orally		
Binahmed^19^	90	302	Post-op: 300 mg penicillin V orally 4 times a day,	0.12% chlorhexidine pre- and postoperative mouth rinses	301/302
			or in the case of penicillin allergy, 150 mg clindamycin		
			orally 3 times a day for 7 days		
Gynther^20^	147	790	Pre-op + post-op: 1g oral	5 mL mouth rinse of povidone-iodine solution (73 mg/mL) was given immediately preoperatively	741/790
			phenoymethyl, 1hr pre-op, and every 8 hrs post-op		
Gynther^20^	132	664	No antibiotics	5 mL rinse of povidone-iodine	630/664
				solution immediately preoperatively	
Laskin^12^	387	1743	Pre-op:Cephalosporin (13% of implants)	0.12% *chlorhexidine*	1662/1743
			Erythromycin (7% of implants) Penicillin (69% of implants)		
			Other (10% of implants)		
Laskin^12^	315	1287	No antibiotics	0.12% *chlorhexidine*	1158/1287
Morris^21^	Not stated	1175	Pre-op: Peterson^1^, AHA (1990)^22^, AHA (1997)^25^	Not stated	1131/1175
Morris^21^	Not stated	354	No antibiotics	Not stated	337/354
Kashani^22^	868	2236	Post-op: 2 g phenoxymethyl	Not stated	2177/2236
			penicillin twice daily for 1 week		
Kashan^22^	868	785	Pre-op + Post-op: 2g phenoxymethyl penicillin	Not stated	777/785
			1 hour pre-op and one dose post-op the same day		
Wagenberg^23^	891	1561	Pre-op + Post-op: Amoxicillin (500 mg 4 times daily)	0.12% cholorhexidine	1515/1561
			2 days pre-op 10 days post-op	3 times daily post-op	
Wagenberg^23^	891	364	Pre-op + Post-op: Clindamycin	0.12% cholorhexidine 3 times daily post-op	333/364
			(300 mg 4 times daily) 2 pre-op, 10 days post-op		

## Results

The results of the literature review showed no significant difference between the success rate of implants with and without the use of antibiotics. Implants performed with the use of antibiotics had a success rate of 96.5% while surgeries performed without antibiotics had a slightly lower success rate of 92%. When pre and post-op antibiotics were compared, success rates of 96% and 97% were found respectively (Table 2). The overall success rate of implants when antibiotics were used was 96.5% and 92% when they were not used (Table 3).

**Table 2:** Post-op Success Rates

**Prophylactic regimen**	**Number of implants**	**Success rate**
No antibiotic	2305	2125/2305 = 92 %
Pre-op antibiotic	3363	3238/3363 = 96%
Post-op antibiotic	2236	2177/2236 = 97%
Both pre and post-op antibiotic	3500	3366/3500 = 96%

**Table 3:** The Overall Success Rate of Implants

	**Total case success with antibiotics used**	**Total case success with no antibiotics used**	**Average comparison between success rates**
Success rate of antibiotic use vs no use	8783/9101	2125/2305	Antibiotics: 96.5% No Antibiotics: 92%

## Discussion

The literature search performed produced an equal division between studies supporting the use of antibiotic prophylaxis and those negating the use of antibiotics. However, a draw back in the current literature became evident as many studies were excluded from this literature review because they did not include comparisons between no antibiotic, pre-op, post-op and both pre and post-op antibiotic use. However, this was a significant finding on its own in that many more studies are required to help validate and improve current guidelines regarding antibiotic use and oral implants.

One of the only commonalities amongst most of the papers was the feeling that antibiotics are overused and that the authors requested that all practitioners’ assess each patient individually in the hopes of reducing the amount of prescribed antibiotics. Of the papers which met the inclusion criterion, 50% agreed that pre- or post operational antibiotics were of benefit while the remaining 50% believed that antibiotics were not of any benefit. With such contrasting results one wonders how these studies could have differed so significantly. Differences may have been inherent within the patient population; healthier patients with stronger immunity versus patients with lower resistance, the number of times patients in studies had been previously prescribed antibiotics, cross-reactions with other drugs, or concomitant illnesses. Unfortunately, this reveals that much of the research completed to date on this topic is inconsistent and lacking in validity. For instance, no randomized controlled studies have been done. In addition, due to the lack of standardization many studies were difficult to compare and had to be excluded. Thus, until larger studies can be completed it will be difficult to determine a definitive answer.

Some alternative methods of lowering the risk of infection that have been explored include the use of Chlorhexidine digluconate (CHX), a mouthwash rinse which is often used in conjunction with dental implants. CHX, when rinsed preoperatively has been proven to be an effective aid in promoting healing and reducing surgical complications [24]. CHX also has been shown to have a high substantively, with the capability to be released over an extended period of time without losing its efficacy. Lambert, et al. (1997) also found that the infectious complications which lead to implant failure were more likely to occur during the closed healing period. Thus, CHX rinse has been shown to be an effective alternative in reducing infectious complications from implant surgery when routinely used in the peri-operative period, and should be used by practitioners who are concerned about infection, if not as the primary means of prevention than at least as an adjunct. Other factors affecting success rates of implants that might be of greater importance include intra-operative management, skill of the surgeon in applying the basic principles of surgery and sanitary conditions, and the patient’s medical status. Early loading of the implant, lack of sufficient alveolar bone, and patient factors such as hygiene levels and the use of alcohol and tobacco all increase the risk of post operative infection [25-29].

After a thorough analysis of the literature, one can conclude that there is no clear evidence pointing to the need for prophylaxis antibiotics in conjunction with dental implant surgery. Of every million patients receiving just a single dose of oral amoxicillin, mild, moderate, and severe allergic reactions have been estimated to occur in 2400, 400, and 0.9 patients, respectively [20]. The dental profession should diligently consider its responsibility of administering antibiotics only when needed, thus avoiding unnecessary allergic reactions whenever possible.

The scientific literature supports the limited use of prophylactic antibiotics, yet clinicians are continually over prescribing them [30]. This non-evidence based practice protocol raises serious ethical concerns. Surgeons and general practitioners alike are routinely placing implants with antibiotics perhaps due to the fact that they are fearful of the legal repercussions of failure. The cost-benefit ratio of any therapy, including all potential adverse effects, must be determined. Studies of this nature with respect to the treatment of infective endocarditis have already been conducted. The ill advised use of antibiotics has proven to be expensive as well as directly responsible for development of resistant microorganisms [7].

The most common adverse effects of antibiotics are direct toxicity, hypersensitivity reactions, and the short or long term development of resistant microorganisms. Direct toxicity includes gastrointestinal (nausea, vomiting, diarrhea, and abdominal pain), hematological concerns (neutropenia, thrombocytopenia, and hemolysis), alterations in the body’s normal flora leading to candidal infections or pseudomembranous colitis, nephrotoxicity (proteinuria or renal failure), neuropathy (nerve dysfunction or peripheral neuropathy), alterations in drug interactions, and finally hepatobiliary (jaundice or hepatitis) [14].

Hypersensitivity reactions can range form mild to lethal. Mild reactions include cutaneous eruptions such as rashes, exfoliate dermatitis, or uticaria. Another complication with antibiotics is known as serum sickness, which is an immune complex condition. The most severe form is an immediate hypersensitivity including anaphylaxis, brochospasms, or laryngeal edema [31].

From a long-term perspective one must be able to appreciate the concerns when a patient develops antibiotic resistant microorganisms. This is a potentially catastrophic concern, which is very difficult to measure. In addition, there is a tremendous financial concern with respect to the development of new drug therapies to treat such patients. The negative effects associated with use of antibiotic therapy must be assessed in comparison to the costs and morbidity related to treating infective endocarditis or infected prosthetic materials. If the risk-benefit and cost-benefit ratios are thoroughly assessed, it becomes clear that if there are specific therapeutic indications based on sound physiologic, anatomic and scientific evidence, then antibiotic prophylactic therapy may be justified [32]. However, many professional associations, i.e., the Academy of Orthopaedic Surgeons (AAOS) [32], American Dental Association (ADA) and the American Heart Assoication (AHA) [17] have written guidelines regarding specific conditions in which it is important to prophylaxe and if these are followed and efforts are made to balance the cost-benefit ratio in patients that fall into the ”grey zone“, then the dental profession can hopefully curb the use of unnecessary antibiotics and keep antibiotic efficacy high for when they are truly necessary.
